# The introduction of risk stratified screening into the NHS breast screening Programme: views from British-Pakistani women

**DOI:** 10.1186/s12885-020-06959-2

**Published:** 2020-05-20

**Authors:** Victoria G. Woof, Helen Ruane, David P. French, Fiona Ulph, Nadeem Qureshi, Nasaim Khan, D. Gareth Evans, Louise S. Donnelly

**Affiliations:** 1 Division of Psychology & Mental Health, School of Health Sciences, Faculty of Biology, Medicine and Health, University of Manchester, MAHSC, Room 1.13, Coupland 1, Coupland Street, Off Oxford Road, Manchester, M13 9PL UK; 2 Nightingale & Prevent Breast Cancer Research Unit, Manchester University NHS Foundation Trust (MFT), Southmoor Road, Wythenshawe, Manchester, M23 9LT UK; 3 NIHR School of Primary Care, School of Medicine, Tower Building, University Park, Nottingham, NG7 2RD UK; 4 Department of Genomic Medicine, Division of Evolution and Genomic Science, MAHSC, University of Manchester, Manchester University NHS Foundation Trust, Oxford Road, Manchester, M13 9WL UK

**Keywords:** UK, Risk stratified screening, Breast cancer risk, Underserved populations, British-Pakistani women, Qualitative interviews

## Abstract

**Background:**

UK national guidelines suggest women at high-risk of breast cancer should be offered more frequent screening or preventative medications. Currently, only 1 in 6 high-risk women are identified. One route to identify more high-risk women is via multifactorial risk assessment as part of the UK’s NHS Breast Screening Programme (NHSBSP). As lower socioeconomic and minority ethnic populations continue to experience barriers to screening, it is important that any new service does not exacerbate issues further. To inform service development, this study explored views of women from underserved backgrounds regarding the introduction of risk stratification into the NHSBSP.

**Methods:**

Nineteen semi-structured interviews were conducted with British-Pakistani women from low socioeconomic backgrounds from East Lancashire, UK. Fourteen interviews were conducted via an interpreter.

**Results:**

Thematic analysis produced three themes. *Attitudes toward risk awareness* concerns the positive views women have toward the idea of receiving personalised breast cancer risk information. *Anticipated barriers to accessibility* emphasises the difficulties associated with women’s limited English skills for accessing information, and their I.T proficiency for completing an online risk assessment questionnaire. *Acceptability of risk communication strategy* highlights the diversity of opinion regarding the suitability of receiving risk results via letter, with the option for support from a healthcare professional deemed essential.

**Conclusions:**

The idea of risk stratification was favourable amongst this underserved community. To avoid exacerbating inequities, this new service should provide information in multiple languages and modalities and offer women the opportunity to speak to a healthcare professional about risk. This service should also enable completion of personal risk information via paper questionnaires, as well as online.

## Background

In 2013 the UK National Institute of Health and Care Excellence (NICE) Clinical Guidelines for familial breast cancer suggested that breast screening should be stratified, with high-risk women (defined as having a ≥ 30% chance of developing breast cancer in their lifetimes or a ≥ 8% 10-year risk) being offered the opportunity for more frequent screening or chemoprevention medication [1]. Similarly for women at above-average (moderate) risk (defined as having a 17 to 29% chance of developing breast cancer in their lifetime), chemoprevention medication and additional screening should be *considered* as preventative treatment and as early detection measures [1].

Currently, most women attending routine mammography are not provided with their personal breast cancer risk estimate, and are not offered risk stratified screening or risk reduction strategies. To fully implement NICE guidelines [1], risk estimation and stratified screening will need to be embedded into routine breast screening programmes to enable *all* women at high or above-average (moderate) risk to benefit from preventative services. Given this, there are efforts globally to promote the introduction of risk stratified screening for breast cancer into national screening programmes [2–6].

It is possible to reliably estimate a woman’s risk of developing breast cancer by combining self-report questionnaire data with mammographic breast density [3, 4, 7, 8]. The addition of Single Nucleotide Polymorphisms (SNPs) also increases the accuracy of this risk estimate [3, 9–11]. A large feasibility study, titled Breast Cancer Predict (BC-Predict) has recently begun in England, aiming to assess the feasibility of offering women their 10-year risk of developing breast cancer as part of routine screening [12]. To calculate risk, BC-Predict is utilising the Tyrer-Cuzick model of risk estimation [7], incorporating mammographic breast density and, in a subsample of participants, SNPs.

The primary aims of feasibility studies like BC-Predict are to assess whether the proposed service can work and whether it is acceptable before piloting [13, 14]. Therefore, it is important to ensure that procedures are acceptable to all women, and do not disadvantage women in minority populations. As part of the BC-Predict feasibility study there is opportunity to highlight and address issues that could arise as a result of implementing risk stratification into routine breast screening programmes. For successful implementation, it is vital that all women of breast screening age (aged 50 to 70 in the UK) who may wish to have their risk calculated feel the service is both acceptable and accessible.

Women from low socioeconomic and minority ethnic backgrounds have low breast screening attendance [15–17]. South Asian women in particular attend breast screening less often than white women and other Black, Asian, Minority Ethnic and Refugee (BAMER) populations [18]. Emotional and cultural barriers are commonly associated with South Asian women’s low attendance at breast screening, as well as language barriers creating significant accessibility issues [18–20]. It is therefore essential that the implementation of risk stratified breast screening does not exacerbate these difficulties further but instead attempts to acknowledge and mitigate them.

When considering the risk of breast cancer in the UK Asian population, it has been found that Asian women (including women of Pakistani and Bangladeshi origin) in Greater Manchester have a lower predictive risk of developing the disease compared to white British/Irish women [21]. Nevertheless the risk of breast cancer in the UK Asian population is expected to steadily rise. This is due to the adverse effects of modifiable risk factors, such as increases in adult weight gain and levels of physical inactivity [21]. To enable *all* women to access preventative interventions to reduce risk, the implementation of risk stratified screening would benefit from being appraised by a population of women whose risk is expected to rise and who also *currently* experience significant barriers to breast screening.

In the present study, qualitative interviews were conducted with British-Pakistani women from East Lancashire, UK. Compared to other communities of Asian women in the UK, British-Pakistani women have particularly low attendance at breast screening [16,22], as well as poorer health outcomes generally [23]. The present research aimed to, (1) explore the views that these women have toward the implementation of risk stratified screening into the NHSBSP and (2) assess the acceptability and accessibility of the BC-Predict study procedures and materials. This report aims to highlight views regarding the intended service and the requirements needed to enable women from underserved populations to access this new programme. Findings from this study would also be used to make amendments to BC-Predict study procedures and materials.

## Methods

### Design

This research employed a cross-sectional design. Face-to face semi-structured interviews were used. This method enables the participant to be open with their responses and allows the researcher to probe areas of interest to answer the research question and aims. Open-ended questions were used flexibly throughout the interviews.

### Procedure

Women were eligible to participate if they were British-Pakistani and from a low socioeconomic background. Socioeconomic background was defined by Index of Multiple Deprivation (IMD) score, calculated by postcode [24]. Women approaching and of screening age were eligible to participate. As there is an age extension trial taking place across England, where women between the ages of 47 to 73 are being invited for breast screening, the research team deemed it appropriate to invite women approaching screening age. Women were excluded if they had a history of breast cancer or if they were not biologically born female. Women were not screened for their fluency in English as professional interpreters were to be arranged. English fluency was not an inclusion requirement as researchers aimed to access views from women who were likely to experience significant difficulties in attending for screening and thus where the introduction of a new service could exacerbate these issues.

Prior to recruitment the research team (NK) approached South Asian women to participate in Patient and Public Involvement (PPI) sessions. Women in these sessions provided advice on executing an effective recruitment strategy and refined the content of the interview schedule. They recommended hosting community events and providing information socially as an effective strategy to generate interest in the study.

The research team organised a community event to engage women from British-Pakistani backgrounds living in East Lancashire, UK. East Lancashire was chosen as the recruitment area as uptake to breast screening is below the targeted 70% [25] and the area has the largest population of British-Pakistanis living in the UK [26]. The community event was advertised via posters (translated into the most common spoken languages in the area), as well as via community outreach workers who were known to women in the area. The aim of the event was to provide information and advice to women about breast screening and establish interested in the present study. At the event, researchers (VGW, LSD & HR) explained the present study and provided women with information sheets and ways of contacting the research team if they were interested in participating. Two interpreters were also present at the event to facilitate communication between the women and researchers.

To further support recruitment, community outreach workers distributed information about the study on the research team’s behalf. Women interested in participating provided the outreach workers with their contact details to enable the research team to arrange an interview. After receiving the contact details of those interested in taking part a member of the research team (VGW) contacted each woman via telephone/telephone interpretation service to answer questions and arrange an interview.

Interviews took place in women’s homes or a private space at a community centre. An NHS approved interpreter assisted in 14 interviews, speaking in either Urdu or Punjabi. The interpreter translated responses in the first and third person and the interviewer addressed the interviewee directly. In one case the interviewee’s daughter was present and translated for her mother alongside the professional interpreter. The remainder of the interviews (5 in total) were conducted in English at the interviewees’ request.

The interviews were split into two phases. In the first phase women were asked about their experiences of breast screening, the challenges they face and their views on the service as a whole. In the second phase of the interviews, presented here, women were asked about risk estimation and risk stratification. To provide women with context the interviewer explained how a woman could obtain a risk estimate as per the BC-Predict feasibility study procedure. The interviewer explained that women would be required to complete an online risk questionnaire, as well as have a mammogram to enable risk to be calculated. Women were then asked about their views on the proposed service. At this stage of the interview women were also shown a prototype information leaflet and risk result letter and asked to comment on these materials (see supplementary files one and two). The interpreter, if needed, translated the materials for the women. All interviews were audio recorded and transcribed verbatim, except one who did not consent to audio recording. Field notes were kept for this interview instead.

### Participants

Nineteen women were interviewed. Interviews lasted one to two hours. All participants were born in Pakistan and moved to the UK with their husbands and families. At the time of interviewing, all women were living in highly deprived areas of East Lancashire, UK. Five women were under 50 years of age, two did not disclose their ages and the remaining women were of breast screening age (50+). All women of breast screening age reported attending for a mammogram at least once.

### Analysis

Data were analysed in NVivo11 using thematic analysis. For this thematic analysis a realist perspective was adopted and researchers analysed the data at a manifest level. As researchers in the second phase of the interviews had specific queries about attitudes toward risk stratification and the accessibility of this new service, themes were developed deductively. However, coding was approached iteratively and inductively, so not to miss nuances in the data. Braun and Clarke’s [27] processes for conducting a thematic analysis were followed flexibly. Two members of the research team (VGW and LSD) began by familiarising themselves with the transcripts through multiple readings. As a realist perspective was applied to this analysis the researchers approached the data with the view that interviewees’ realities are communicative through the dataset. Initial coding was conducted by VGW and codes became more refined following the analysis of each transcript. Regular meetings were had between members of the research team (VGW, LSD, HR and FU) to discuss the initial codes and themes. Codes and themes continued to be refined in this manner and assessed for their representativeness across the dataset. The final thematic structure was achieved following this process. Recruitment was stopped and data sufficiency achieved when there appeared to the researchers (VGW, LSD, HR and FU) to be no new content being discussed in the final two interviews.

## Results

Views from British-Pakistani women regarding the introduction of risk stratified screening into the NHSBSP were grouped into three themes: (1) Attitudes toward risk awareness, (2) Anticipated barriers to accessibility, and (3) Acceptability of risk communication strategy. All quotes have been anonymised and participants assigned pseudonyms. Findings relating to phase 1 of these interviews (i.e. women’s experiences and views of breast screening) are reported in a corresponding publication [28].

### Theme 1: attitudes toward risk awareness

All participants unanimously indicated that they would want to know their personal risk of developing breast cancer in the next ten years. There were no participants who were against learning their risk. Women explained that risk information is important for healthcare professionals to share and introducing this service into the NHSBSP would be a good idea in order to raise awareness:

*Yes, you should do, should give out this. It is a good idea to let people know whereabouts they stand on the risk band. Because anything to do with your health and wellbeing, it's good to have that information available so that you can make improvements and adjustments in order to improve that risk factor. (Fatima, 60, via interpreter)*

Participants expressed the view that providing risk information would allow women to be more aware of the impact that developing breast cancer could have on their lives, enabling them to take positive action to prevent the disease from developing:

*They should be told, people should be aware, whether they want to know about it, or they don’t [..], they should have this awareness of how it’s spreading so much that you could be a target, to avoid that you should get all this done. And if you’re a high risk, you can prevent it from not happening to you… (Sadia, 60, via interpreter)*

Women reacted positively to the lifestyle information presented in the information materials, explaining that preventative information was valuable in order to be clear on how to reduce risk and be healthy. For some women, knowing their risk would also encourage them to continue attending for routine mammograms:

*If I get a letter like I am an average, say, I will be aware about that I have a risk and really that means I’m not a below average so I have to be careful, I think then I need to go if they invite me again. (Yarsa, 41, via interpreter)*

### Theme 2: anticipated barriers to accessibility

Barriers to accessibility focused on the problems that could be caused by having to provide self-report information via an online questionnaire. The modality chosen to communicate risk results and risk information was also discussed in appreciation of accessibility. When discussing in what modality the risk questionnaire would be provided, the majority of women explained that online completion could be problematic. Many were not computer literate and a paper questionnaire printed in their own language would be a preferable alterative, ‘*She’s not tried anything on the computer, so she wouldn’t know. For her it would be better if it was a letter form’ (Roshanah, 50,* via *interpreter).* Some women explained that should online questionnaire completion be the only option, they would need to rely on family members, specifically daughters and daughters-in-law to assist them. A distinction was made between different generations of women, with older women said to be more reliant on family member input:

*If it’s the older generation, they would just get some help from their daughters, daughter in laws, get that support, and tell them the answers, and they fill in, and that way they could do it. But, the generation below that are fairly educated, in a sense, where they can do it themselves, it’s not a problem. (Bushra, no age provided, via interpreter)*

Women also explained that all information, including risk letters, would be more accessible if printed in their first languages. This would enable them the autonomy to read and understand the information for themselves, rather than receive second-hand translations via family members or healthcare professionals:

*…it’s better in my own language because then I can understand better. Sometimes I have a medicine so I ask my doctor or my husband please read for me what is the side effect. They say oh don’t worry, it’s okay. So other person don’t pay attention with you... (Namra, no age provided)*

Women acknowledged that if materials could not be automatically provided in their spoken language, the option to request translated versions would be beneficial. However, providing translations would not make materials accessible for all, with many of the women identifying that not all women in their communities are literate in the languages they speak.

### Theme 3: acceptability of risk communication strategy

Women had mixed opinions on the acceptability of receiving a personalised risk result via letter. The acceptability of the approach was dependent upon the severity of the result. Women were concerned that a high-risk result received via letter would cause distress. They felt that without the immediate opportunity to ask questions, women might speculate as to what a high-risk result would mean for their health. In this instance it was suggested that a face-to-face consultation with a healthcare professional would be more suitable:

*She said someone with a high-risk, it would be quite scary and they might have so many questions that are buzzing through their head then at that point. So it would be better really for someone in that position who's in a higher risk to be able to be told face-to-face. (Fatima, 60, via interpreter)*

For women with limited literacy skills, receiving a high-risk result letter written in English could cause additional concerns due to their inability to access the information directly:

*If that lady who got the letter, she doesn’t know English, she doesn’t understand English that would be a worry if they got the letter first and then somebody might, on behalf of her, talk to the doctor. (Sara, 41)*

In some cases, rather than sending a personalised risk result directly to women’s homes it was deemed more appropriate for their General Practitioner (GP) to receive this information first. Women explain that as a high-risk result is likely to cause significant worry, talking with a healthcare professional in the first instance would provide reassurance and alleviate concerns. Some acknowledged that they would prefer their GP to notify them directly if they were found to be at high-risk:

*She thinks the best way to do it, is not send a letter home, basically send it to the GP. If the letter goes straight to the doctor, and the doctor does this and tells them, there’s trust obviously with the doctor. (Roshanah, 50, via interpreter)*

In contrast some women described that receiving results via letter is the most efficient communication strategy, in spite of the level of risk. Alternatives, such as receiving a telephone call or arranging a GP appointment were viewed as problematic:

*Personally, I think a letter is a good thing, because it’s not easy to go to see the GP you know? You have to take time, you can’t make an appointment nowadays, it’s very hard. (Ifrah, 70 via interpreter)*

Women who advocated communicating risk via letter still emphasised the importance of being able to contact a professional should they have questions. It was therefore suggested that letters should explicitly encourage all risk groups to get in contact with a healthcare professional should they have concerns.

## Discussion

Views toward implementing risk stratified routine breast screening were positive, with British-Pakistani women wanting to know their personal risk of developing breast cancer. Similar positivity toward individual risk assessment for breast cancer has also been observed in underserved women in the US [29, 30]. Despite this, several issues that implementation would need to address were highlighted, including the accessibility of the service and the acceptability of risk result communication. Potential difficulties to access were emphasised, with language barriers affecting knowledge acquisition and online questionnaire completion presenting a significant challenge. The acceptability of providing women with their risk result via letter was also deliberated, with some women believing that the delivery of a high-risk result should be made via a trusted healthcare professional (i.e. their GP). However others advocated receiving personal risk information via letter, providing the option to speak with a healthcare professional (HCP) is available.

Strengths of this study included recruiting a particular underserved population in the UK, all of whom were living in low socioeconomic areas and from British-Pakistani backgrounds. By having PPI involvement and establishing trust in the community, an effective recruitment strategy was developed. This enabled the research team to engage women who are often described as ‘hard-to-reach’ and thus underrepresented in the literature [31]. Furthermore, instead of women providing feedback based upon a hypothetical proposal of risk stratification [32, 33], this study was able to gain informed insights by providing women with a model of how risk stratification could be integrated into routine breast screening.

Despite the strengths of this study, there were limitations that should be acknowledged. As the women interviewed were all literate in either English, Punjabi or Urdu, the perspectives of those who have limited to no literacy skills were lacking. Although this challenge *was* acknowledged by the women in this study, further research should aim to access views about risk stratified breast screening from those who experience significant language difficulties. Additionally, there were limited discussions in the interviews regarding the language used to describe risk stratified screening. For example, knowledge and understanding of complex terms, such as ‘breast density’ and ‘chemoprevention medication’ were not addressed. Given that this cohort of women identified difficulties in understanding terms such as, ‘screening’ and ‘mammogram’ [28], it is likely that similar issues would be experienced with risk stratification terminology, yet this was not explicitly discussed here.

The women in the present study described that knowing risk and receiving risk personalised estimates would provide motivation for continued engagement with breast screening. Participants stipulated that increased engagement with breast screening would likely be driven by increased awareness about being at high or moderate risk. This is in contrast to a study, by Schwartz et al. (1999) whereby breast cancer risk counselling reduced mammography adherence in women with limited education and a family history of breast cancer [34]. Attitudes toward preventive lifestyle information presented via the information leaflet was favourable in this study, with women describing the advice as invaluable for attaining control over their risk. These views are also reflected in an interview study conducted in the US, where low-income African American and Latina women at increased risk of breast cancer felt empowered to seek knowledge in order to prevent the development of the disease [30]. However, more recent evidence suggests that communication of future risk is unlikely to result in behaviour change [35, 36].

The acceptability of delivering a personalised risk result via letter was questioned, with understanding the results and effect on wellbeing being of concern. There is evidence that the emotional impact of receiving breast cancer risk estimates is minimal for most women at low to moderate risk [35]. In the present study adverse emotional effects from receiving risk results via letter were thought to be most relevant for women at high-risk. These findings are in line with survey data exploring preferences for notification methods for communicating breast cancer risk results [37, 38]. In these studies participants preferred to receive face-to-face communication if results suggested that they were at high-risk of breast cancer [36, 37]. Nevertheless, evidence still remains mixed as to whether a test result delivered via letter for cancer screening contributes to increased anxiety [39]. However, for participants in this study, English language proficiency and an inability to understand information in the letter were identified as unique factors which could heighten anxiety, especially if the letter communicated a high-risk result, and this is likely to be true for other Ethnic populations receiving risk estimates in English as opposed to their first language.

In the present study we have shown that despite whether women advocate receiving risk results via letter, all women expressed the need to have questions answered by a HCP. This reflects the view that combined written and verbal communication could enhance women’s understanding of risk results and mitigate any induced anxiety [39]. Women in the present study highlighted their GP as a HCP to whom they could trust to explain their results. The role of GPs in breast cancer risk communication is reflected in a recent systematic review of 50 studies exploring women’s views of risk based screening and prevention for breast cancer [40]. In this review GPs were identified as the preferred HCP to have conversations with women about chemoprevention [40].

## Implications for practice and future research

To facilitate access to risk stratified screening the translation of information materials into a variety of languages will be required. Should a complete translation not be possible, key health messages should be provided in women’s preferred languages to aid accessibility and understanding. The inclusion of information which enables women to request information in a different language or format could also prove beneficial, providing that instructions on how to do so are given in the most common languages spoken. The present study has shown that paper copies of the self-report risk questionnaire will need to be available, with their availability clearly advertised. Moreover, should women at high and above-average (moderate) risk wish to receive a telephone call or attend a risk consultation, the opportunity to have an interpreter present should be detailed in their risk result letter. Provisions for women who have limited literacy skills will need to be explicitly managed. Consultations between HCPs and service users who have limited reading and writing skills would be beneficial to establish an optimal way for these women to access this new service.

Furthermore, regular events should be hosted in areas of familiarity to women across the community, i.e. faith community centres and schools. To aid accessibility, it would be helpful if appropriately trained bilingual lay health workers were employed at these events. The employment of lay health workers has been successful amongst BAMER groups for improving uptake to breast, cervical and bowel screening programmes [41]. These community events could prove particularly helpful for women with limited literacy skills both in English and their spoken languages. Nevertheless, for women who have difficulty reading and writing, presentation at community events may be challenging, as often individuals chose not to disclose their literacy difficulties [42]. It is important to note that low literacy levels is not just a challenge for BAMER groups, with 1 in 6 adults in England described as having poor literacy skills [43]. Therefore, efforts should be made to provide information about risk stratified screening in alternative formats for all women. For BAMER populations, cultural and language sensitive telephone interventions to increase breast screening uptake have been successful in the UK [44]. However further research should continue to engage all women with low literacy skills to explore the modalities in which risk communication could be better disseminated.

Opinions as to whether a risk result should be communicated via a letter were mixed amongst the present sample of women. Some women identified that they would prefer their GP to notify them personally if they were at high-risk of breast cancer. How practical and feasible it would be for GPs to provide risk feedback in this way requires exploration. Research across primary and secondary care is currently taking place to access HCP’s views on the introduction of risk stratified breast screening into the NHSBSP. This research will provide GPs with the opportunity to detail how they envisage their involvement and thus how feasible it would be for them to provide risk feedback to women in the manner described in the present study. However in spite of whether GPs have a designated role in the implementation of risk stratified breast screening, it is still likely that all women would contact primary care as a ‘first port of call’ to have their questions answered. As the present study suggests, women from BAMER communities may especially wish to speak with their GP. Therefore whatever their level of involvement primary care would still require training and support to manage these queries.

In spite of risk level, all women in the present study were unanimous with regards the importance of having access to HCP support. Presently in the BC-Predict feasibility study women at high and above-average (moderate) risk are given the opportunity to speak with a breast cancer risk expert to discuss preventative treatment options. This offer is communicated via the risk result letter. Should those at high and above-average (moderate) risk continue to receive notification of their results via letter, consultation information should not only be framed as a chance to discuss preventative treatments but also as an opportunity to have questions answered and concerns alleviated. Thus indicating to women that access to support is available and encouraged. As women in the present study described HCP support as an important feature of this new service, information regarding how to access this support will need to be communicated in various formats and languages. For all women and for all risk groups to feel supported, a variety of information and support options, including a designated contact and telephone hotline (with translation support) would be beneficial.

## Conclusions

To aid access for BAMER women to risk stratified screening in the NHSBSP, this service will need to be cognisant of language barriers by producing information materials in common spoken languages, facilitating interpreter support if required and producing physical copies of risk questionnaires. The feasibility of all risk groups receiving contact from a HCP is questionable, given that 2.23 million women in the UK attend for breast screening each year [45]. Nevertheless, the opportunity for an appointment with a trained HCP in risk communication should be strongly encouraged for any woman at high and above-average (moderate) risk. Arguably, frontline HCPs involved in relaying breast cancer screening results, such as GPs, should develop the skills to communicate risk to all women, including those from diverse backgrounds. To be supported in doing so, GPs will require training and information materials to help them effectively deal with queries from women. Women should also be made aware of designated pathways and contacts should they want to discuss and receive further information about risk. In consideration of future research, the area of risk stratified breast screening would benefit from more research which addresses women’s attitudes, knowledge and understanding of risk, risk calculations and preventative treatments.

## Supplementary information

**Additional file 1.**

**Additional file 2.**

## Data Availability

The dataset analysed is not publicly available as it may contain information that would compromise participant consent. Contact the corresponding author for more information.

## Abbreviations

NICE: National Institute of Health and Care Excellence
NHSBSP: NHS Breast Screening Programme
SNPs: Single Nucleotide Polymorphisms
BAMER: Black, Asian, Minority Ethnic and Refugee
PPI: Patient and Public Involvement
GP: General Practitioner
HCPs: Healthcare Professionals
