# Supplementary material for: The introduction of risk stratified screening into the NHS breast screening Programme: views from British-Pakistani women
Source: BMC Cancer. 2020 May 20;20:452. doi: 10.1186/s12885-020-06959-2 (PMC7240981; doi:10.1186/s12885-020-06959-2)
Supplement: Supplementary file 1 — Additional file 1. [file 12885_2020_6959_MOESM1_ESM.pdf]

University Hospital of South Manchester NHS Foundation Trust

PROCAS Study

Reducing your risk of breast cancer

This leaflet is designed to accompany your risk feedback letter

You took part in a research study run by the Genesis and Nightingale Centre at the University Hospital of South Manchester. This study is called Predicting Risk of Cancer at Screening (or PROCAS for short).

PROCAS is looking into ways to predict breast cancer. For this, we assessed your risk of developing breast cancer in the next 10 years.

Background

Breast cancer is the most common type of cancer in the UK.

In 2011, 49,936 women were diagnosed with breast cancer between the ages of 15 and 99 years. Of these 78% (3 out of 4) are predicted to live for 10 or more years after diagnosis.

This high rate of survival is in part due to cancers being detected at an earlier stage. The earlier a cancer is detected, the more effectively it can be treated.

Many things contribute to your chance of developing breast cancer. Some are more preventable and some are less preventable. More detail about breast cancer risk factors can be found on the following page. Your risk has been based on these factors.

Breast cancer risk factors

Less preventable risk factors include:

- Age
- Family history
- Breast density
- Never having had children
- Being 30 years or older at the birth of your first child
- Going through menopause after the age of 55
- Starting your periods before the age of 12

Preventable risk factors include:

- Being overweight
- Drinking alcohol
- Being physically inactive
- Smoking

Age

The older you are the greater your chances of developing breast cancer. Around four out of five breast cancers occur in women aged 50 and over.

Hormones and reproduction

The female sex hormones, oestrogen and progesterone, can affect the development of breast cancer.

- Hormone replacement therapy (HRT) increases the risk of breast cancer. The risk associated with HRT is reduced 5 years after you stop taking it.
- Oral contraceptives (also known as the Pill) increase the risk of breast cancer but can reduce the risk of ovarian and womb cancers. The risk associated with the Pill is reduced 10 years after you stop taking it.
- Starting your periods at a younger age or having a late menopause increases the risk of breast cancer.
- Having children and breast feeding both lower the chances of developing the disease. The more children a woman has, and the younger she is when she has them, the lower her risk.

Family history

Women who have relatives with breast cancer have a higher risk of developing the disease themselves.

- Risk increases with the number of close relatives diagnosed especially if they were diagnosed at a younger age.
- But even so, about eight out of ten breast cancers occur in women with no close relatives diagnosed with the disease.

Reducing your risk of breast cancer

Maintain a healthy weight

Being overweight and gaining weight throughout adult life increases the risk of developing breast cancer after the menopause. Try to maintain a healthy weight by combining a balanced diet, including plenty of fruit and vegetables, with regular physical activity.

Exercise regularly

Women who are physically active are less likely to develop breast cancer than non-active women. Try to do at least 150 minutes of moderate physical activity a week, such as 30 minutes of walking five times a week. The more active you are, the more you can reduce your risk. For advice on how to lose weight and increase physical activity, consult your GP practice or see the NHS Choices website at: http://www.nhs.uk/livewell

Limiting alcohol

Drinking alcohol raises the risk of breast cancer. For overall health you don't need to cut alcohol out altogether. Try to keep to sensible intakes of less than 14 units week, or 2-3 units/ day ensuring you have at least 2 alcohol free days/ week. 14 units is slightly less than a bottle and a half of wine/ week or 10 measures of spirit/ week. For advice on reducing your alcohol intake, please see the NHS Change4Life website at: www.nhs.uk/Change4Life

Not smoking

Some research suggests that smoking increases the risk of breast cancer. For advice on stopping smoking, contact your GP or join an NHS Stop Smoking Service. You can find your local Stop Smoking Service through the website www.nhs.uk/smokefree or call the Smokefree National Helpline to speak to a trained adviser on 0800 0224 332.

Drug prevention

Women at above average (moderate) risk can ask their GP for more information about taking a drug (tamoxifen or raloxifene) to reduce their risk of breast cancer.

Signs and symptoms of breast cancer

If you get to know how your breasts normally look and feel, you will be more likely to spot any changes that could be signs of breast cancer. This is important, even if you have been for breast screening. Look out for the following:

• A lump or thickening in the breast

• A change in the nipple. The nipple might be pulled back into the breast, or change shape. You might have a rash that makes the nipple look red and scaly, or have blood or another fluid coming from the nipple.

• A change in how your breasts feel or look. They may feel heavy, warm or uneven, or the skin may look dimpled. The size and shape of the breast may change.

• Pain or discomfort in the breast or armpit.

• A swelling or lump in the armpit

Even women with a below average risk can still develop breast cancer, so it is important to know the signs of breast cancer and what you can do to reduce your risk.

If you have any changes to your breast, you should make an appointment to see your GP straight away. You may not have cancer. But if you do, being diagnosed and treated at an early stage makes it more likely that you would survive breast cancer.

If you have a family history of breast cancer you should discuss this with your GP who can refer you to a Family History Clinic to discuss your risk further.

If you have any questions about screening, you can contact your GP or the Greater Manchester Breast Screening service on 0161 291 4444.

You may find the following websites helpful in telling you more about breast cancer and ways to reduce your risk.

Genesis Breast Cancer Prevention: www.genesisuk.org
Cancer Research UK: www.cancerresearchuk.org
Macmillan Cancer Support: www.macmillan.org.uk
Breast Cancer Care UK: www.breastcancercare.org.uk
Breast Cancer Now: www.breastcancernow.org

For further details regarding the PROCAS study, please contact:

PROCAS team
Genesis and Nightingale Prevention Centre
University Hospital of South Manchester
Wythenshawe Hospital
Southmoor road,
Manchester
M23 9LT

Phone: 0161 291 4409
E-mail: PROCAS.study@UHSM.NHS.UK

This information was developed by the breast cancer prevention research team from the Nightingale and Genesis Breast Cancer Prevention Centre at the University Hospital of South Manchester (UHSM), with funding from Genesis Breast Cancer Prevention Appeal Ltd.

PROCAS risk feedback leaflet: v2.0 24th July 2015
Review date latest: July 2016

genesis preventing breast cancer

UHSM Your Hospital
