# Supplementary material for: The introduction of risk stratified screening into the NHS breast screening Programme: views from British-Pakistani women
Source: BMC Cancer. 2020 May 20;20:452. doi: 10.1186/s12885-020-06959-2 (PMC7240981; doi:10.1186/s12885-020-06959-2)
Supplement: Supplementary file 2 — Additional file 2. [file 12885_2020_6959_MOESM2_ESM.docx]

#
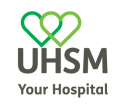

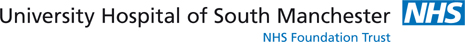


**NIHR Programme Grant BREAST CANCER PREDICTION and PREVENTION**

Professor Anthony Howell

Nightingale Centre &

The Genesis Prevention Centre

Wythenshawe Hospital

Southmoor Road

Wythenshawe

Manchester, M23 9LT

Tel: 0161 291 4409

Email: [PROCAS.study@uhsm.nhs.uk](mailto:PROCAS.study@uhsm.nhs.uk)

Professor Gareth Evans

Dr Susan Astley

ADDRESS:

DATE:

Dear **[INSERT NAME]**,

**RE: PROCAS study – Predicting Risk of Cancer at Screening**

**NHS number:**

Thank-you for taking part in the PROCAS study. As part of that study, when you attended your mammogram on **[INSERT DATE]**, we asked you to complete a questionnaire where you gave us some background information. Your risk is calculated from a combination of factors which are outlined in the accompanying leaflet. We have calculated your risk of developing breast cancer in the next 10 years from the information you have provided us with on the questionnaire and your breast density (the amount of tissue in your breast that is not fat) as assessed from your mammogram.

Your risk of developing breast cancer in the next 10 years was calculated to be **above average (moderate) risk.** This means that out of 100 women with risk factors like you, between 5 and 7 women will develop breast cancer **within 10 years of that mammogram.**

**High**

8 to 26 women out of 100 will develop breast cancer.

**74 – 92 of those women will NOT develop the disease.**

**Above average (moderate)**

5 to 7 women out of 100 will develop breast cancer.

**93-95 of those women will NOT develop the disease.**

**Average**

2 to 4 women out of 100 will develop breast cancer.

**96-98 of those women will NOT develop the disease.**

**Below average**

0 to 1 women out of 100 will develop breast cancer.

**99 of those women will NOT develop the disease.**

**Within 10 years**:

**High**

**8 or more**

**women out of 100 will develop breast cancer**

**Above average**

5-7 women out of 100 will develop breast cancer

**Average**

2-4 women out of 100 will develop breast cancer

**Below average**

0-1 women out of 100 will develop breast cancer

**High**

**8 or more**

**women out of 100 will develop breast cancer**

**Above average**

5-7 women out of 100 will develop breast cancer

**Average**

2-4 women out of 100 will develop breast cancer

**Below average**

0-1 women out of 100 will develop breast cancer

**High**

**8 or more**

**women out of 100 will develop breast cancer**

**Above average**

5-7 women out of 100 will develop breast cancer

**Average**

2-4 women out of 100 will develop breast cancer

**Below average**

0-1 women out of 100 will develop breast cancer

**High**

**8 or more**

**women out of 100 will develop breast cancer**

**Above average**

5-7 women out of 100 will develop breast cancer

**Average**

2-4 women out of 100 will develop breast cancer

**Below average**

0-1 women out of 100 will develop breast cancer

There are things that all women can do to reduce their risk of breast cancer, such as losing weight (if needed) through diet and exercise. Being overweight increases the risk of developing breast cancer. If you are overweight, losing at least 5% of your weight and keeping it off can reduce your risk of breast cancer by around 25%, even if breast cancer is in your family. Losing weight can also help reduce your risk of developing other diseases, such as heart disease, diabetes and dementia. More information on the ways to reduce your risk together with signs and symptoms of breast cancer are given in the accompanying leaflet.

**If you want to discuss you risk further, please make an appointment with the study team on 0161 291 4408 who will arrange either a telephone consultation or face-to-face appointment at your local Family History Clinic.** During this appointment, your breast cancer risk will be explained to you along with information on the ways in which to reduce your risk that you may be eligible for, such as taking a drug (tamoxifen or raloxifene) for prevention.

In summary, you have an increased risk of developing breast cancer compared to other women in Manchester. Please remember that 93-95 women out of 100 women in your risk group will **not** develop breast cancer in the next 10 years.

Yours sincerely,

Professor Gareth Evans Paula Stavrinos

Professor of Medical Genetics Study Co-ordinator

**If you think your risk estimate is not accurate**

The risk estimate is based on the information you gave to us when you joined the study. Risk may change over time, however as this is a research study and risk assessment is not yet part of routine clinical care, unfortunately we are unable to re-calculate your risk for you.

If you have concerns that your risk may have increased significantly and you think you may be eligible for referral to a family history clinic, please see your GP.

**If you have been diagnosed with breast cancer**

The speed at which we receive information about who has been diagnosed with breast cancer depends on which hospital you were diagnosed at. If you have been diagnosed with breast cancer since joining the study, please contact us on 0161 291 4409 and we will be able to send you a more relevant letter.

**Queries about your breast screening appointments**

If you have a query regarding breast screening, please call 0161 291 4444, or email: bsapts@uhsm.nhs.uk
